# PYY-Dependent Restoration of Impaired Insulin and Glucagon Secretion in Type 2 Diabetes following Roux-En-Y Gastric Bypass Surgery

**DOI:** 10.1016/j.celrep.2016.03.091

**Published:** 2016-04-21

**Authors:** Reshma D. Ramracheya, Laura J. McCulloch, Anne Clark, David Wiggins, Helene Johannessen, Magnus Kringstad Olsen, Xing Cai, Chun-Mei Zhao, Duan Chen, Patrik Rorsman

**Affiliations:** 1 Oxford Centre for Diabetes, Endocrinology and Metabolism, University of Oxford, Oxford, Oxon OX3 7LE, UK; 2 Department of Cancer Research and Molecular Medicine, Norwegian University of Science and Technology, 7491 Trondheim, Norway; 3 Metabolic Research, Department of Physiology, Institute of Neuroscience and Physiology, University of Goteborg, Box 432, 40530 Göteborg, Sweden

## Abstract

Roux-en-Y gastric bypass (RYGB) is a weight-reduction procedure resulting in rapid resolution of type 2 diabetes (T2D). The role of pancreatic islet function in this restoration of normoglycemia has not been fully elucidated. Using the diabetic Goto-Kakizaki (GK) rat model, we demonstrate that RYGB restores normal glucose regulation of glucagon and insulin secretion and normalizes islet morphology. Culture of isolated islets with serum from RYGB animals mimicked these effects, implicating a humoral factor. These latter effects were reversed following neutralization of the gut hormone peptide tyrosine tyrosine (PYY) but persisted in the presence of a glucagon-like peptide-1 (GLP-1) receptor antagonist. The effects of RYGB on secretion were replicated by chronic exposure of diabetic rat islets to PYY in vitro. These findings indicate that the mechanism underlying T2D remission may be mediated by PYY and suggest that drugs promoting PYY release or action may restore pancreatic islet function in T2D.

## Highlights

• Roux-en-Y gastric bypass rapidly restores islet function and morphology in diabetic GK rats
• The effects of RYGB on islet function are mediated by the gut hormone PYY and not GLP-1
• In vitro PYY application to diabetic islets restores insulin and glucagon secretion

## Graphical Abstract

**Figure:**
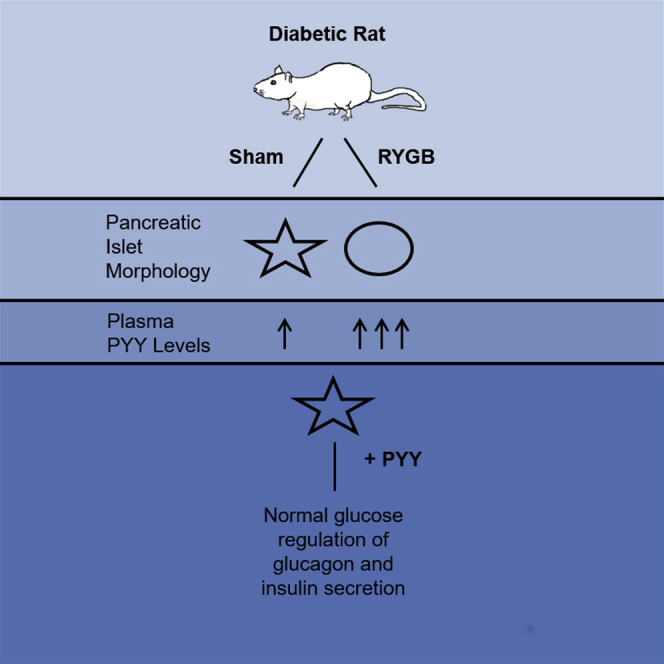

## Teaser

RYGB rapidly reverses diabetes. Ramracheya et al. demonstrate that RYGB normalizes glycaemia in diabetes by restoring insulin and glucagon secretion. They show that the gut hormone PYY mediates these improvements and that it restores secretory function in diabetic islets. These findings suggest that PYY might be a T2D therapeutic target.

## Introduction

Roux-en-Y gastric bypass (RYGB) is the most common form of weight loss surgery. Although developed originally as a weight-reduction therapy, RYGB can lead to full and durable remission of type 2 diabetes (T2D) in up to 90% of cases (Buchwald et al., 2004). The remission occurs within days of surgery and before any significant weight loss. The mechanisms behind this improvement remain unknown.

Human T2D is characterized by dysfunction of both glucose-stimulated insulin secretion (GSIS) from pancreatic β cells and inappropriate regulation of glucagon production from α cells (Unger and Cherrington, 2012). The importance of glucagon in diabetes is confirmed by the finding that mice lacking glucagon receptors remain normoglycemic even after complete destruction of β cells (Lee et al., 2012).

Although pancreatic islets are center stage in diabetes, the impact of RYGB on islet function, especially on glucagon secretion, has not been fully elucidated. Elevated levels of the incretin hormone GLP-1 were initially thought to trigger improved glucose homeostasis and remission of diabetes upon surgery. However, the beneficial effects of surgery persist in rodents lacking both GLP-1 and its receptor, suggesting a GLP-1-independent mechanism (Mokadem et al., 2014, Ye et al., 2014).

Using a diabetic rat model of RYGB, we have investigated the effects of surgery on islet function and the underlying mechanisms.

## Results

### RYGB Restores Impaired Islet Secretory Properties and Improves Metabolic Parameters

**Figure 1:**
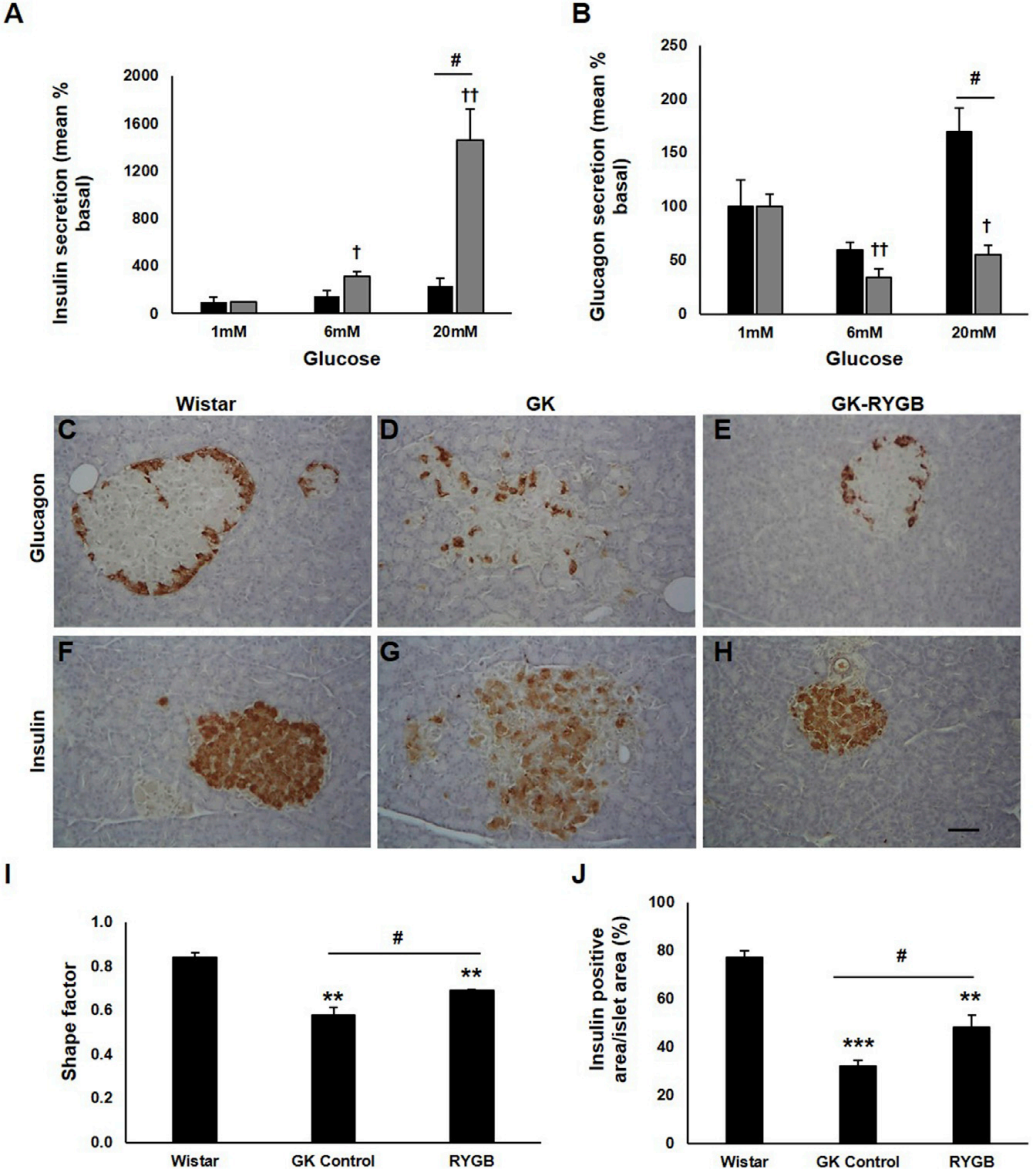
Islet Secretion and Morphology Are Improved in GK Rats Post-RYGB (A and B) Insulin (A) and glucagon (B) secretion in islets isolated from sham (black bars) or RYGB (gray bars) animals. Secretion is presented as percentage of that secreted at basal (mean ± SEM as percentage of total islet content; insulin: sham, 0.023 ± 0.004; RYGB, 0.046 ± 0.001; glucagon: sham, 0.216 ± 0.053; RYGB, 1.314 ± 0.025; n = 6–12 rats; two to five experiments). ^†^p < 0.05, ^††^p < 0.01 versus 1 mM glucose, ^#^p < 0.05 for indicated comparisons. (C–H) Pancreatic sections from Wistar (C and F), GK control (D and G), and GK RYGB (E and H) animals, stained for glucagon (C–E) and insulin (F–H). Scale bar represents 100 μM. (I) Shape factor analysis, used as a measure of islet structure, was determined in GK controls (n = 4), Wistar (n = 3), and RYGB-operated animals (n = 3). (J) Insulin positive area/total islet area was assessed in these animals (n > 30 islets/animal). Data are presented as mean ± SEM, ^∗∗^p < 0.01, ^∗∗∗^p < 0.001 compared to Wistar controls, ^#^p < 0.05 for indicated comparisons. See also Figures S1 and S2.

Both insulin and glucagon secretion was assessed in islets isolated from spontaneously diabetic Goto-Kakizaki (GK) rats post-RYGB surgery. Islets from the sham-operated diabetic group displayed reduced GSIS and lack of glucose-induced suppression of glucagon release, which was stimulated rather than inhibited at 20 mM glucose (Figures 1A and 1B). These abnormalities are strikingly similar to those observed in islets from T2D patients (Zhang et al., 2013).

RYGB surgery resulted in marked improvement of GSIS compared to the sham operation (the response at 6 and 20 mM glucose increased 1.4-fold and 12.7-fold, respectively) (Figure 1A). Glucagon suppression was restored at 6 and 20 mM glucose in the RYGB group (Figure 1B), and the inverted glucose response observed at 20 mM glucose in the sham group was corrected. In addition, there was a significant increase in insulin (RYGB versus sham, 13.1 ± 1.0 versus 6.9 ± 0.8 ng/islet; mean ± SEM) and glucagon content (RYGB versus sham, 894.2 ± 37.6 versus 630.2 ± 69.7 pg/islet) in the RYGB group. RYGB animals lost 8% of their body weight compared to 1% in the sham-operated group (Figure S1A). This was associated with a modest reduction in calorie intake (Figure S1B), a significant decrease in plasma glucose levels (Figure S1C), and a 4-fold drop in water consumption in the RYGB animals (Figure S1D), consistent with remission of diabetes.

### RYGB Influences Islet Gene Transcription

The effects of RYGB on islet secretion correlated with changes in gene expression levels. Expression of insulin, glucagon, and somatostatin genes were elevated ∼5-, 3-, and 9-fold, respectively, in the RYGB group compared to sham animals (Figure S2A). Moreover, expression of *Pdx1* and *Nkx6.1*, two key transcription factors regulating β cell development and identity (Cerf, 2006), were elevated 3- and 4-fold, respectively.

### RYGB Restores Islet Morphology

Whereas islets from non-diabetic Wistar rats are largely spheroidal (Figures 1C and 1F), GK rat islets exhibit a “starfish” appearance (Höög et al., 1997) (Figures 1D and 1G). Following RYGB, islet morphology was restored to that of non-diabetic islets (Figures 1E and 1H). The morphological changes were quantified by islet shape factor measurements (Figure 1I). Analyses of immuno-labeled sections demonstrated that the proportion of insulin-positive cells/islet was lower in the sham-operated group compared to RYGB rats. Insulin-positive cell proportions were significantly higher following RYGB (Figure 1J) and the intra-islet localization of the glucagon-positive α cells was similar to that seen in non-diabetic islets (compare Figures 1C and 1E). There was no change in the α cell area proportion (Figure S2B). Islet density/pancreas was lower in diabetic rats, a factor that was not corrected by RYGB (Figure S2C).

### Effects of RYGB on Islet Function Are Modulated by a Humoral Factor

**Figure 2:**
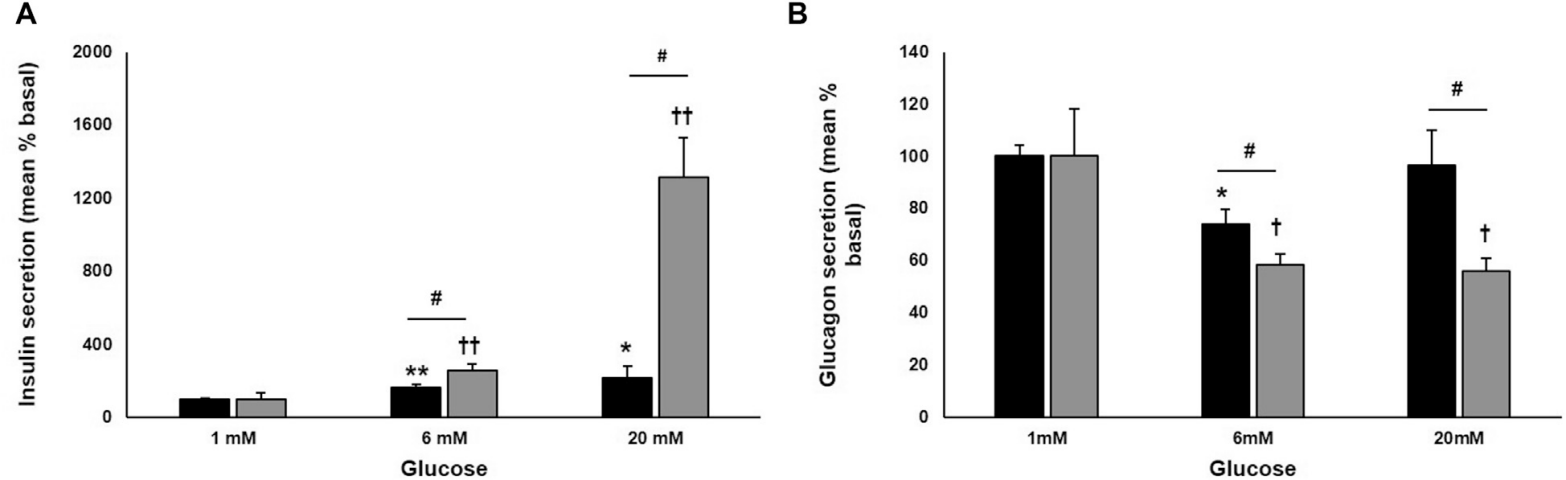
Metabolic Benefits of RYGB Are Driven by a Humoral Factor (A and B) Insulin (A) and glucagon (B) secretion from rat islets treated for 48 hr with serum from sham- (black) or RYGB-operated (gray bars) animals. Data are presented as percentage of basal secretion (mean ± SEM as percentage of content; insulin: sham, 0.974 ± 0.064; RYGB, 2.274 ± 0.0749; glucagon: sham, 0.748 ± 0.032; RYGB, 5.251 ± 0.947; n = 8 rats, five or six experiments). ^∗^p < 0.05, ^∗∗^p < 0.01 versus 1 mM glucose sham, ^††^p < 0.01 versus 1mM glucose RYGB, ^#^p < 0.05 for indicated comparisons. See also Figure S3.

To determine whether the effects of RYGB on pancreatic islet function was related to a blood-borne factor, rat islets were pretreated for 48 hr with sera obtained from sham or RYGB rats. Whereas islets treated with sham serum exhibited only a 2-fold increase in GSIS, islets exposed to serum from RYGB animals displayed a 14-fold stimulation (Figure 2A). Glucagon suppression at 6 and 20 mM glucose was significantly improved by treatment with RYGB serum (Figure 2B). Insulin and glucagon content were significantly higher in the RYGB serum-treated group compared to the sham serum group (Figures S3A and S3B).

### Effects of RYGB Are Not Mediated by GLP-1

**Figure 3:**
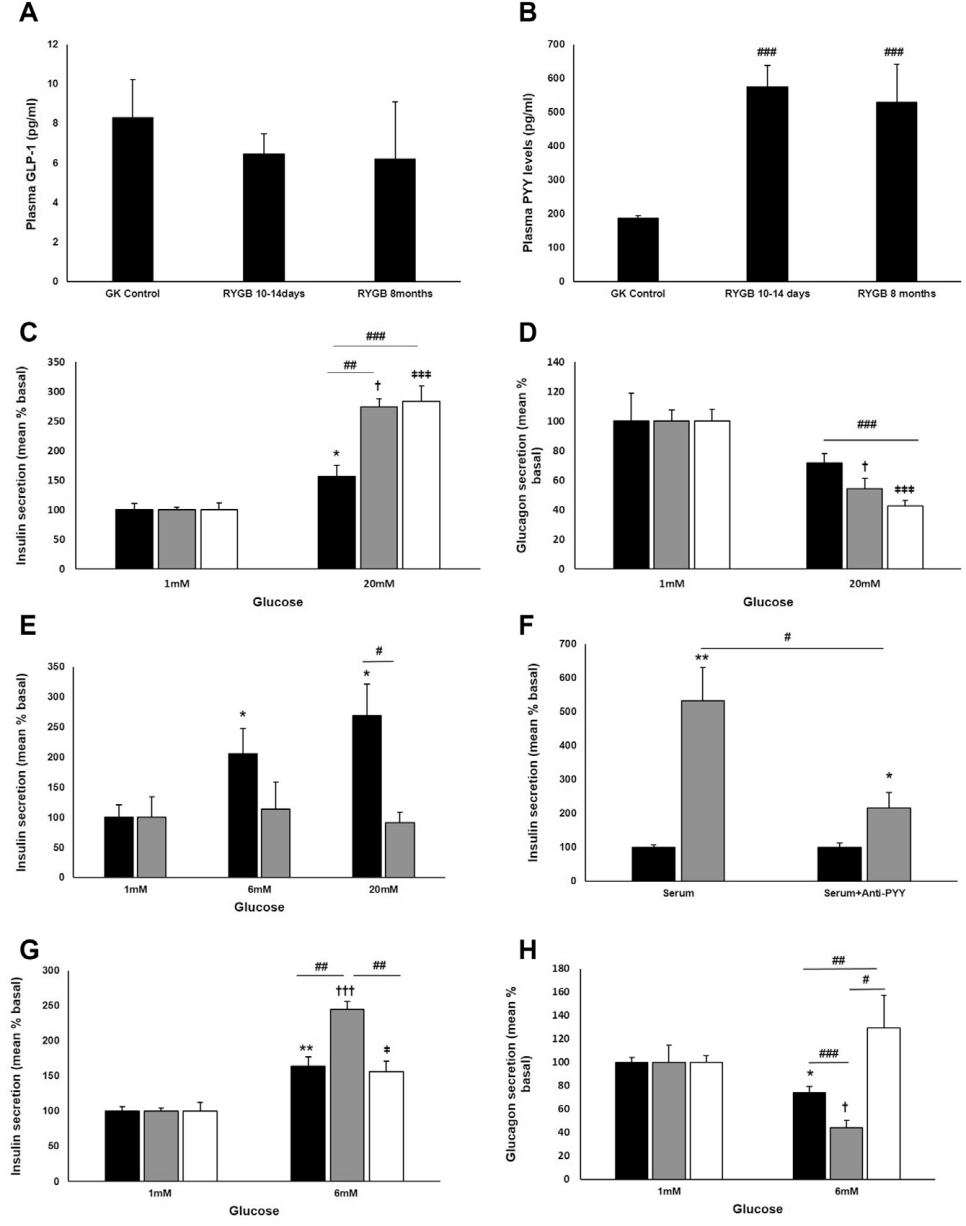
PYY Mediates the Effects of RYGB (A and B) Total plasma GLP-1 (A) and PYY (B) levels in GK sham-operated and RYGB animals at 10–14 days and 8 months post-surgery. (C and D) Insulin (C) and glucagon (D) secretion from rat islets chronically cultured with 100 pM PYY (gray), 100 nM PYY (white), or in the absence of PYY (black bars). Data are presented as percentage of basal secretion (mean ± SEM as percentage of content; insulin: 100 pM PYY: 0.1 ± 0.004; 100 nM PYY: 0.137 ± 0.016; control alone: 0.133 ± 0.014; glucagon: 100 pM PYY: 2.731 ± 0.211; 100 nM PYY: 2.287 ± 0.0.185; control alone: 1.196 ± 0.368; n = 6–8 rats; 3–11 experiments). ^∗^p < 0.05 versus 1 mM glucose control, ^†^p < 0.05 versus 1 mM glucose with 100 pM PYY, ^ǂǂǂ^p < 0.001 versus 1 mM glucose with 100 nM PYY, ^##^p < 0.01, ^###^p < 0.001 for indicated comparisons. (E) Insulin secretion from rat islets chronically cultured with PYY in the presence (gray) or absence (black) of the *NPY1R* antagonist BIBP-3226 (1 μM). Data are presented as percentage of basal secretion (mean ± SEM as % of content; PYY: 0.062 ± 0.013; PYY+BIBP-3226: 0.178 ± 0.061; n = 6 experiments). ^∗^p < 0.05 versus 1 mM glucose control, ^#^p < 0.05 for indicated comparison. (F) Insulin secretion from rat islets at 1 mM (black) and 20 mM (gray bars) glucose following chronic exposure to RYGB serum, with or without PYY antibody (1/500). Data are presented as percentage of basal secretion (mean ± SEM as % of content; serum alone: 0.223 ± 0.018; with antibody: 0.253 ± 0.03; n = 6 experiments). ^∗^p < 0.05, ^∗∗^p < 0.01 versus 1 mM glucose. (G and H) Insulin (G) and glucagon (H) secretion from rat islets following chronic exposure to sham-operated serum supplemented with 100 nM PYY with (white) or without (gray) PYY antibody (1/500) or sham-operated serum alone (black). Data are presented as percentage of basal secretion (mean ± SEM as % of content; insulin: PYY with antibody: 0.009 ± 0.001; PYY without antibody: 0.006 ± 0.0002; sham serum alone: 0.974 ± 0.064; glucagon: PYY with antibody: 0.472 ± 0.026; PYY without antibody: 1.109 ± 0.0.161; sham serum alone: 0.748 ± 0.032; n = 6–8 rats; six experiments). ^∗^p < 0.05, ^∗∗^p < 0.01 versus 1 mM glucose sham serum, ^†^p < 0.05, ^†††^p < 0.001 versus 1 mM glucose sham serum with PYY, ^ǂ^p < 0.05 versus 1 mM glucose sham serum, PYY and anti-PYY. ^#^p < 0.05, ^##^p < 0.01, ^###^p < 0.001 for indicated comparisons. See also Figures S3 and S4.

Increased post-prandial GLP-1 levels have been implicated as the causal factor for improved glucose homeostasis and remission of diabetes following RYGB (Lindqvist et al., 2013), but it is notable that the beneficial effects of surgery persist in mice lacking GLP-1 and its receptor (Mokadem et al., 2014, Ye et al., 2014). We investigated the involvement of GLP-1 in the RYGB GK rat model. Enhanced GSIS in islets from RYGB animals persisted in the presence of the GLP-1 receptor antagonist exendin (9–39), excluding a role for locally released GLP-1 as a mediator of improved islet function (Marchetti et al., 2012) (Figure S3C). To assess further the possible involvement of systemic GLP-1, plasma levels of the incretin were determined under basal conditions. No significant change in total GLP-1 levels was noted in the RYGB rat serum at 10–14 days or 8 months post-surgery (Figure 3A). Co-application of exendin (9–39) to rat islets pre-cultured with RYGB serum also failed to suppress enhanced GSIS responses (Figure S3D).

### Increased PYY Levels Post-RYGB

Another hormone co-secreted by the L-cells in response to glucose intake is peptide tyrosine tyrosine (PYY) (Adrian et al., 1985). Circulating PYY levels are drastically reduced in obesity and T2D (Batterham et al., 2003). In the RYGB rat model, total plasma PYY concentrations were markedly higher within 10–14 days following surgery compared to the sham group and remained significantly elevated for up to 8 months (Figure 3B).

### Effects of PYY on Islet Secretory Function

Despite an established role of PYY in metabolism, there is a lack of consensus on its role on insulin secretion (Persaud and Bewick, 2014). Moreover, the effect of PYY on glucagon release is unknown. Short-term (1 hr) exposure of rat isolated islets to exogenous PYY did not affect GSIS (Figure S4). To replicate the sustained elevation of PYY seen in vivo, long-term effects of the peptide were studied at a physiological concentration of 100 pM and a higher concentration of 100 nM in vitro using a chronic (48–60 hr) culture protocol. Compared to uncultured islets, this culture protocol is associated with reduced GSIS and loss of glucose-induced suppression of glucagon release. However, treatment with both low and high PYY concentrations resulted in a dramatic improvement of GSIS (Figure 3C) and restoration of glucose-induced inhibition of glucagon secretion (Figure 3D). PYY acts via neuropeptide Y (NPY) family of receptors, of which Npy1r is expressed abundantly in islets at the transcript (Persaud and Bewick, 2014) and protein levels (L.J.M., unpublished data). Inhibition of Npy1r using the antagonist BIBP-3226 resulted in complete reversal of the restorative effects of long-term PYY exposure on GSIS (Figure 3E). Application of the antagonist on its own had no effect on GSIS (Figure S4B).

### Effects of RYGB Are Mediated by PYY

To test whether PYY is the humoral mediator underlying the beneficial effects of RYGB on islet function, healthy rat islets were exposed to serum from RYGB rats in the absence or presence of a PYY-specific antibody at a concentration known to react with the peptide. Immuno-neutralization of PYY attenuated the effects of the serum resulting in a 75% lower response to GSIS compared to that in the absence of the antibody (Figure 3F). The specificity of the antibody was confirmed by pre-incubation of islets with the synthetic peptide, resulting in a lack of immunohistochemical detection of PYY (Figure S4B). When serum from sham-operated rats was supplemented with PYY, it reproduced the beneficial effects of RYGB on islet secretion (Figures 3G and 3H). These effects were reversed by immuno-neutralization of exogenous PYY (Figures 3G and 3H). It is of note that, in contrast to the effects of PYY in the supplemented sham serum, immuno-neutralization failed to fully reverse the effects of RYGB serum (Figure 3F), probably implicating the existence of additional factor(s).

### PYY Restores Impaired Islet Secretion in Diabetes

**Figure 4:**
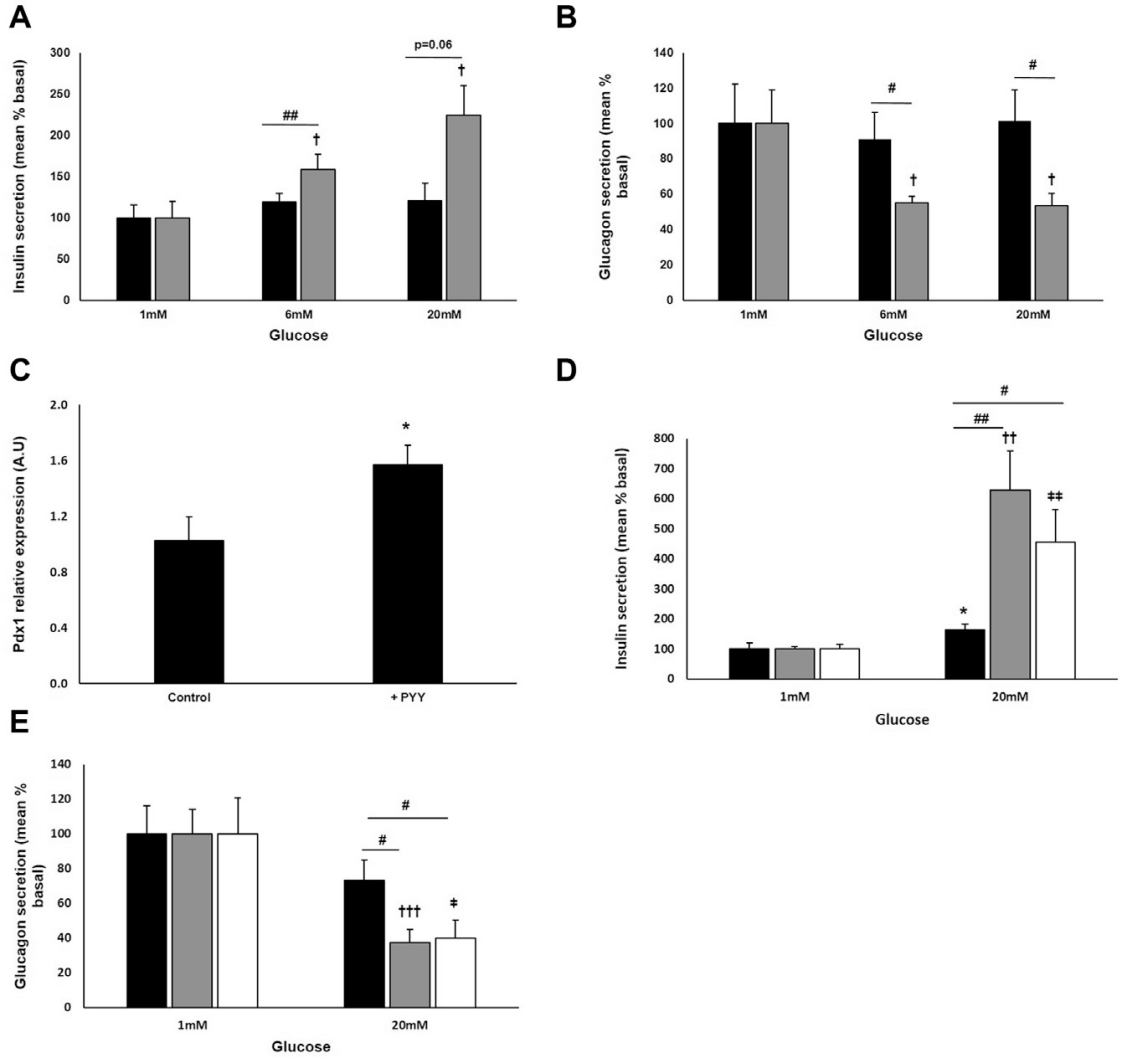
PYY Restores Function in GK Islets and Improves Human Islet Function (A and B) Islets from diabetic GK rats were chronically cultured in the presence (gray) or absence (black bars) of PYY before insulin (A) and glucagon (B) secretion were determined. Data are presented as percentage of basal secretion (mean ± SEM as % of content; insulin: PYY: 0.075 ± 0.015; without PYY: 0.139 ± 0.022; glucagon: PYY: 1.422 ± 0.271; without PYY: 1.443 ± 0.321; n = 5–9 experiments). ^†^p < 0.05 versus 1 mM glucose with PYY, ^#^p < 0.05, ^##^p < 0.01 for indicated comparisons. (C) mRNA expression of the β-cell transcription factor, *Pdx1* was determined post-culture with PYY in islets of diabetic GK rats. Data are presented as mean ± SEM, ^∗^p < 0.05. (D and E) Insulin (D) and glucagon (E) secretion from human islets chronically cultured with 100 pM PYY (gray), 100 nM PYY (white), or in the absence of PYY (black bars). Data are presented as the percentage of that secreted at 1 mM glucose (mean ± SEM as percentage of content; insulin: 100 pM PYY: 0.063 ± 0.004; 100 nM PYY: 0.119 ± 0.018; control: 0.19 ± 0.036; glucagon: 100 pM PYY: 3.023 ± 0.429; 100 nM PYY: 4.137 ± 0.848; control: 1.178 ± 0.189 (n = 14–17 and 17–29 experiments respectively; five or six separate donors). ^∗^p < 0.05 versus 1 mM glucose without PYY, ^††^p < 0.01, ^†††^p < 0.001 versus 1 mM glucose with 100 pM PYY. ^ǂ^p < 0.05, ^ǂǂ^p < 0.01 versus 1 mM glucose with 100 nM PYY. ^#^p < 0.05, ^##^p < 0.01 for indicated comparisons.

PYY infusion in diabetic rats lowers body weight and HbA1c levels (Pittner et al., 2004), but its impact on impaired islet function remains unknown. We tested the effects of PYY directly on islets isolated from severely diabetic GK rats (plasma glucose: 26 ± 2 mM). These islets exhibited impaired glucose-mediated insulin and glucagon secretion (Figures 4A and 4B). However, following treatment with PYY for 48–60 hr, GSIS was restored (Figure 4A) and glucose-induced inhibition of glucagon secretion was normalized (Figure 4B). The changes in islet hormone release were associated with a 1.5-fold increase in *Pdx1* gene expression level (Figure 4C), consistent with the effect of RYGB (Figure S2C).

### PYY Is Effective in Human Islets

Finally, we confirmed that PYY is also effective in human islets. Following chronic treatment with both low and high concentrations of PYY, insulin secretion was enhanced at 20 mM glucose (Figure 4D). Glucose-induced glucagon suppression was also significantly improved in PYY-treated islets (Figure 4E).

## Discussion

RYGB surgery leads to rapid resolution of T2D but the mechanisms involved remain elusive. It is commonly believed that improved islet function underlies the beneficial effects, but mechanistic studies are still missing.

Although increased post-prandial GLP-1 secretion following RYGB surgery has been reported (Salehi et al., 2011, Shin et al., 2010), no changes in fasting GLP-1 levels in RYGB-operated patients have been documented (Clements et al., 2004, Pournaras et al., 2010). Moreover, there is evidence demonstrating that central or peripheral GLP-1 receptor signaling is not critical for the beneficial effects of RYGB. Thus, the role of GLP-1 as a mediator of surgery-associated benefits remains inconclusive. In our study, total GLP-1 levels following RYGB were unchanged in plasma taken under ad libitum feeding conditions.

T2D is a bihormonal disorder and hyperglycemia occurs as a result of inappropriate secretion of both insulin and glucagon (Unger and Cherrington, 2012). We now report that RYGB leads to restoration of both GSIS and glucose-induced suppression of glucagon secretion and that the effects are mediated by a humoral factor. We also demonstrate that this humoral factor is PYY and that it acts via Npy1r. Elevated levels of serum PYY following bariatric surgery has been documented (Chan et al., 2006), but whether this translates into improved islet function has not been studied previously. Our results are consistent with the report that RYGB fails to improve glucose tolerance in PYY-deficient mice (Chandarana et al., 2013). Moreover, PYY ablation has been reported to cause gross impairment of pancreatic β cell structure and function (Sam et al., 2012). Our findings of elevated *Pdx1* expression level in PYY-treated diabetic islets and increased insulin-positive cells following RYGB suggest that PYY may be important in the restoration of β cell functional identity.

Improved islet function following PYY pre-treatment in human islets suggests that it is possible to extend our observations in diabetic rats to man. In humans, circulating PYY occurs in the range of 60 to 100 pM and close to 500 pM in some tissues (Adrian et al., 1985). Our results indicate that PYY is effective in vitro at both physiological (100 pM) and pharmacological (100 nM) concentrations. Acutely administered PYY infusion in obese, healthy volunteers has no effect on plasma insulin (Tan et al., 2014). However, it should be noted that PYY does not affect GSIS when applied acutely but that chronic exposure of islets to PYY restores normal glucose regulation of insulin and glucagon secretion. This time frame is broadly consistent with the beneficial effect of RYGB on glucose homeostasis observed clinically (Buchwald et al., 2004). Chronic administration of PYY to diabetic rats has been shown to result in improved glycemic control (Pittner et al., 2004), but the effects on pancreatic hormone release were not explored. This study demonstrates that within 10–14 days of RYGB surgery, pancreatic islet structure and function in diabetic GK rats are significantly improved. Further studies are warranted to address whether these beneficial changes are sustained over the long term.

Modulation of incretin secretion (for example GLP-1) by bacterial metabolites has been reported (Chimerel et al., 2014). It is therefore likely that changes in gut microbiome following RYGB (Tremaroli et al., 2015) also affect PYY release, which, like GLP-1, is secreted by the entero-endocrine L-cells. A pharmacological agent stimulating PYY production and/or mimicking its action could provide an effective and nonsurgical therapy for T2D.

## Experimental Procedures

### Animals

Adult male Wistar and GK rats (Taconic) were used. Rats were kept in Makrolon Type-4 individually ventilated cages with free access to tap water and standard rat pellet food (RM1 811004). Animals were divided into RYGB or sham-operation group. Age- and sex-matched animals were used as controls. Body weight was recorded daily during the first week after surgery and then weekly throughout the experiments. Experiments were approved by the Norwegian National Animal Research Authority.

### Roux-en-Y Gastric Bypass Model

Animals were operated under anesthesia (isoflurane, 4% for induction and 2% for maintenance). The surgery was performed through a small midline abdominal incision. The intestine was transected 10 cm distal to the ligament of Treitz, creating a distal and a proximal end. The proximal end was anastomosed to the intestine, 15 cm distal to where it was previously transected. A gastric pouch was created (2%–3% of total stomach), and the distal end of the intestine was anastomosed to it in an end-to-side fashion (Kodama et al., 2013). For sham operation, the animals were opened through a midline incision and the viscera were gently manipulated.

### Measurements of Plasma Concentrations of PYY and GLP-1

Blood was drawn from the abdominal aorta at sacrifice under ad-libitum feeding conditions and processed as described previously (Jørgensen et al., 2013). Plasma was kept at −80°C until determination of hormones. Total PYY was measured by radioimmunoassay (Millipore). Total GLP-1 was assayed using the highly sensitive total GLP-1 (v2) kit (K150JVC-1, Mesoscale Discovery).

### Islet Isolation

Animals were used for experiments 10–14 days following surgery. All experiments were conducted in accordance with the UK Animals Scientific Procedures Act (1986). Rat islets were isolated by collagenase type V (Sigma-Aldrich) digestion as described previously (Zhang et al., 2013). Human pancreases were obtained with ethical approval and clinical consent from nondiabetic donors. Islets were isolated in the Diabetes Research & Wellness Foundation Human Islet Isolation Facility by collagenase digestion (Serva) using modified versions of published protocols (Lake et al., 1989). Experiments were performed using islets from five or six separate donors with the following parameters: mean ± SEM; age: 41.7 ± 3.7 years; BMI: 25.8 ± 1.6; islet purity: 66.3% ± 5.5%; islet viability: 77% ± 5.2%.

### Secretion Studies

Islets were used for secretion studies within 2 hr of isolation. Experiments were set with 13–15 hand-picked and size-matched islets per tube (in triplicate). Islets were pre-incubated in Krebs-Ringer buffer (KRB) containing 2 mg/ml BSA and 3 mmol/l glucose for 1 hr at 37°C, followed by another hour of test incubation in KRB supplemented with glucose as indicated. Insulin measurements were performed at 20 mM glucose, but in some cases, to maximize glucagon inhibition from the same islets, stimulated secretion was performed at 6 mM glucose (Ramracheya et al., 2010). Insulin and glucagon content was determined by radioimmunoassay (Millipore). To allow comparison of experiments, secretion data are presented as mean percent basal, where basal is secretion at 1mM glucose.

### Serum and Chronic PYY Exposure Studies

Serum samples from sham-operated and RYGB GK and Wistar rat models were used. Islets were pre-cultured for 48–60 hr in RPMI (5 mM glucose) with the addition of 20% pooled serum or PYY (1–36) or PYY (3–36) (100 pM or 100 nM, Bachem). Both PYY analogs produced the same effects. To explore the role of PYY and GLP-1, anti-PYY antibody (AB22663, Abcam) or BIBP-3226 (1 μM; Tocris) and exendin (9–39) (1 μM; Bachem) were co-applied with the sera or PYY treatment respectively, for 48–60 hr.

### Histology

Pancreases from Wistar (n = 3), sham-operated GK (n = 4), and RYGB GK rats (n = 3) (10–14 days post-surgery) were fixed in 4% formalin, paraffin embedded, and cut into 5-μm sections. Following dewaxing and rehydration, endogenous peroxidase activity was blocked using 0.3% H_2_O_2_. Sections were incubated overnight at 4°C with either mouse anti-glucagon (1:500, Sigma), guinea pig anti-insulin (1:500, in-house), or rabbit anti-somatostatin (1:500, Santa Cruz) and visualized with 3,3′-diaminobenzidine tetrahydrochloride (DAB) (SigmaFast, Sigma-Aldrich). Imaging for morphometric analysis was performed on images using an Axio Vision Imaging System (Zeiss). Shape factor analysis was derived from the area and perimeter of each individual islet (4 × area × π/perimeterˆ^2^), with a perfect circle having a shape factor of 1.

### Statistical Methods

Statistical analyses were performed using Excel. All data are presented as mean ± SEM. Statistical significance was evaluated by Student’s t test or ANOVA analysis. A p value of less than 0.05 was considered significant.

## Author Contributions

D.C., H.J., M.K.O., X.C., and C.-M.Z. planned and performed the animal experiments. R.D.R., D.W., L.J.M., and A.C. performed subsequent experiments and analyzed data. R.D.R., L.J.M., D.C., and P.R. designed the study and wrote the paper. All authors contributed to discussion of the results and preparation of the final manuscript.

## Supplemental Information

Document S1. Supplemental Experimental Procedures and Figures S1–S4

Document S2. Article plus Supplemental Information
