# Supplementary material for: PYY-Dependent Restoration of Impaired Insulin and Glucagon Secretion in Type 2 Diabetes following Roux-En-Y Gastric Bypass Surgery
Source: Cell Rep. 2016 Apr 21;15(5):944–50. doi: 10.1016/j.celrep.2016.03.091 (PMC5063952; doi:10.1016/j.celrep.2016.03.091)
Supplement: Document S1. Supplemental Experimental Procedures and Figures S1–S4 [file mmc1.pdf]

Cell Reports, Volume *15*

Supplemental Information

PYY-Dependent Restoration of Impaired Insulin and Glucagon Secretion in Type 2 Diabetes following Roux-En-Y Gastric Bypass Surgery

Reshma D. Ramracheya, Laura J. McCulloch, Anne Clark, David Wiggins, Helene Johannessen, Magnus Kringstad Olsen, Xing Cai, Chun-Mei Zhao, Duan Chen, and Patrik Rorsman

Supplemental Experimental Procedures

Measurements of food and water intakes and blood glucose levels

Rats were placed in the Comprehensive Laboratory Animal Monitoring System (Columbus Instruments International, Columbus, USA) with free access to standard rat powder food (RM1 801002) and tap water. Parameters that were obtained during daytime (7 am–7 pm) and night time (7 pm–7 am) for each individual rat included number of meals, meal size, meal duration, accumulated food intake, intermeal interval, rate of eating, satiety ratio, drinking activity, energy expenditure and ambulatory activity. Blood glucose was measured after 16 hours fasting in blood from the tail vein and analysed with a glucose meter (FreeStyle Freedom Lite, Abbot Diabetes Care Inc. Alameda, CA, USA).

RNA extraction and processing

Total RNA was extracted from rat islets using the phenol-chloroform-guanidinium-thiocyanate method (Chomczynski and Sacchi, 1987). Briefly, 100 islets were homogenised in Tri Reagent (Life Technologies, Paisley, UK) before RNA isolation using chloroform and isopropanol precipitation. RNA quantity was determined spectrophotometrically using Nanodrop technology. Turbo DNase (Life Technologies) was used to remove any residual genomic contamination before 1µg of RNA was reverse transcribed in a random primed single-strand synthesis reaction.

Gene expression analysis

Gene expression analysis was performed using Taqman technology. cDNA was diluted 1:10 using 0.01M Tris-HCl before amplification with inventoried gene specific assays (primers available on request) or assays against the housekeeping genes *Hprt1* and *B2m*. All samples were amplified in triplicate using gene expression mastermix (Life Technologies) and run in parallel with a standard curve to permit determination of assay efficiency. Amplification was performed using an ABI7900HT thermalcycler at 95°C – 10 min, 50 cycles (95°C – 15 s, 60°C – 1 min). Gene expression levels were calculated using the $2^{\wedge -\Delta\Delta CT}$ method modified by Pfaffl (Pfaffl, 2001) and are presented in arbitrary units (au).

SUPPLEMENTAL DATA ITEMS

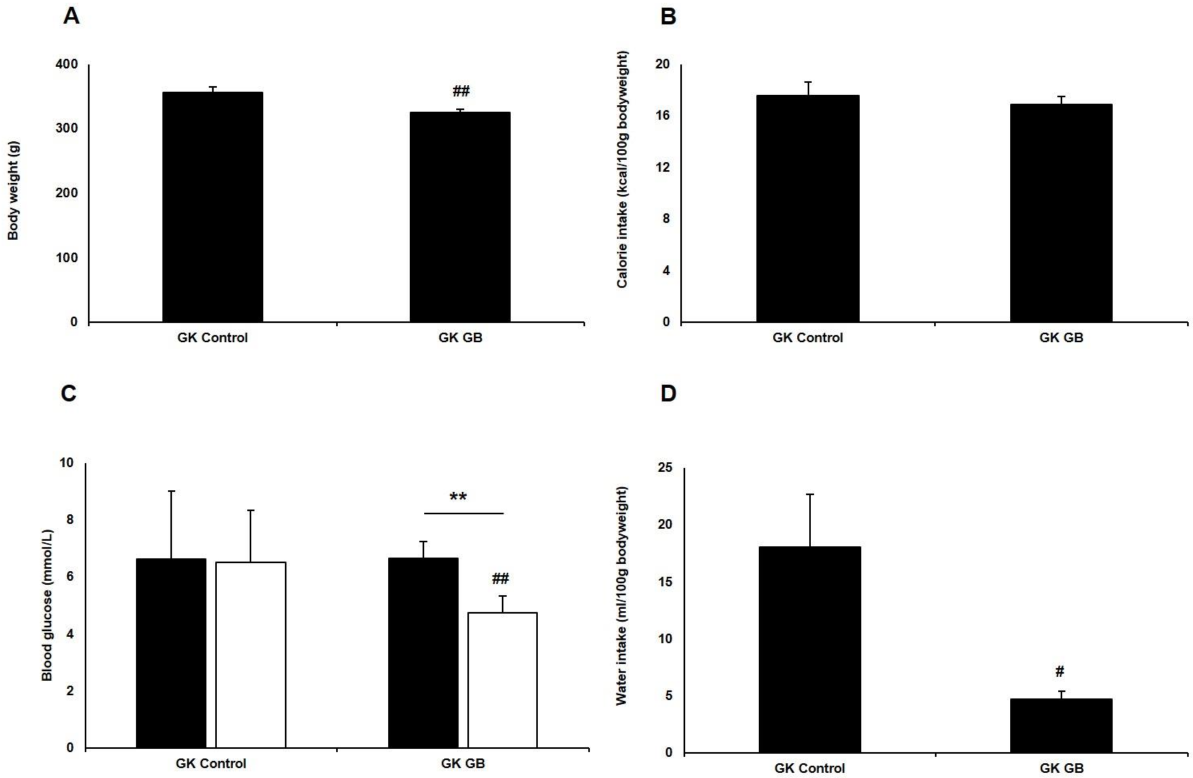

Figure S1. Related to Figure 1. Metabolic health is improved in GK rats following RYGB.

The effects of RYGB on body weight **(A)**, caloric intake **(B)**, blood glucose **(C)** and water intake **(D)** were evaluated 10-14 days post-surgery in bypass and sham-operated animals. GK Control = Sham-operated, GK GB = RYGB. Blood glucose levels are shown at time of surgery (black bars) and 10-14 days post-surgery (white bars). $^{\#}P$<0.05, $^{\#\#}P$<0.01 vs controls. $^{**}P$<0.01 vs pre-surgery. Data represented as mean values ± SEM.

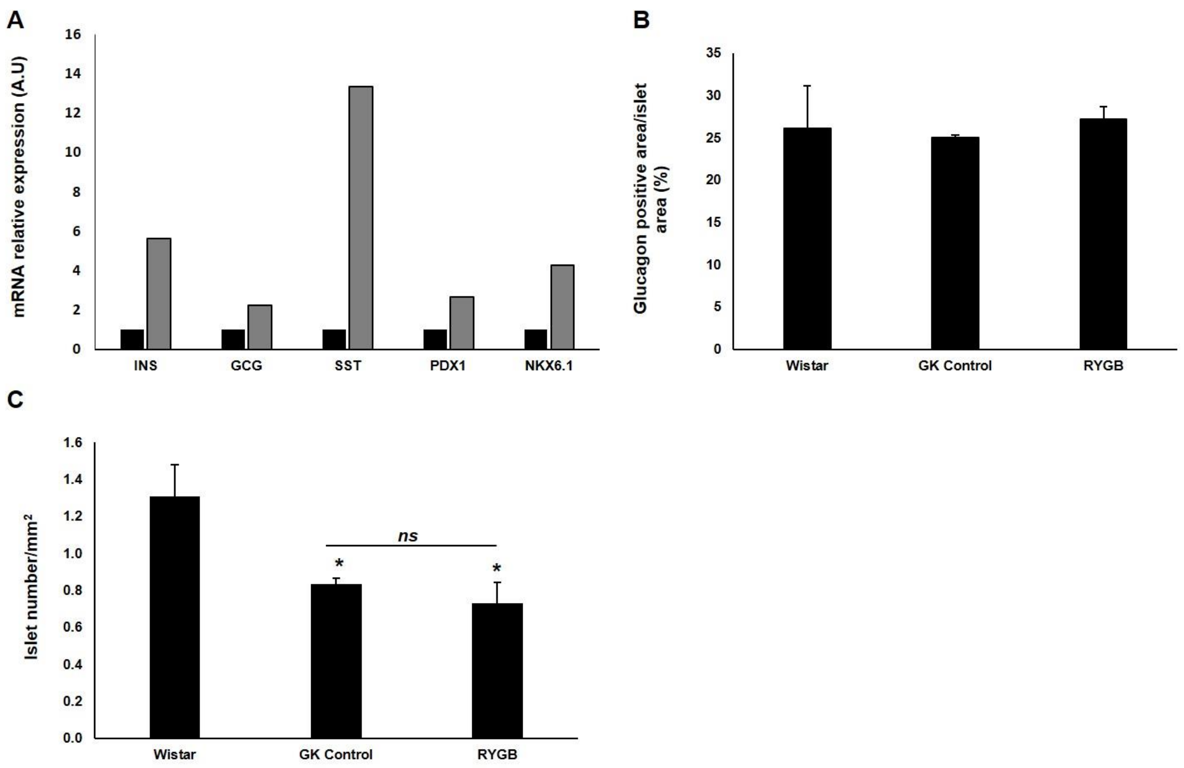

Figure S2. Related to Figure 1. Islet gene expression and morphology post-RYGB.

(A) mRNA expression levels of the pancreatic hormones insulin, glucagon, and somatostatin, and the beta-cell transcription factors *Pdx1* and *Nkx6.1* were determined in islets from sham-operated (black) and RYGB GK rats (grey bars) 10-14 days post-surgery. Islets were pooled from >4 animals to generate a single pool per group. Samples were run in triplicate and mean expression presented in arbitrary units (a.u). Expression in controls (sham-operated) is taken as unity and data in RYGB islets is expressed relative to control values.

(B-C) Islet morphology was studied in sections cut from wax-embedded pancreases of healthy Wistar (n=3), sham-operated GK (n=4) and RYGB GK (n=3) animals. **(D)** Glucagon positive area/Total islet area was assessed in >30 islets per animal. **(E)** Islet number per pancreas was also determined. **P*<0.05 vs Wistar controls. Data represented as mean ± SEM.

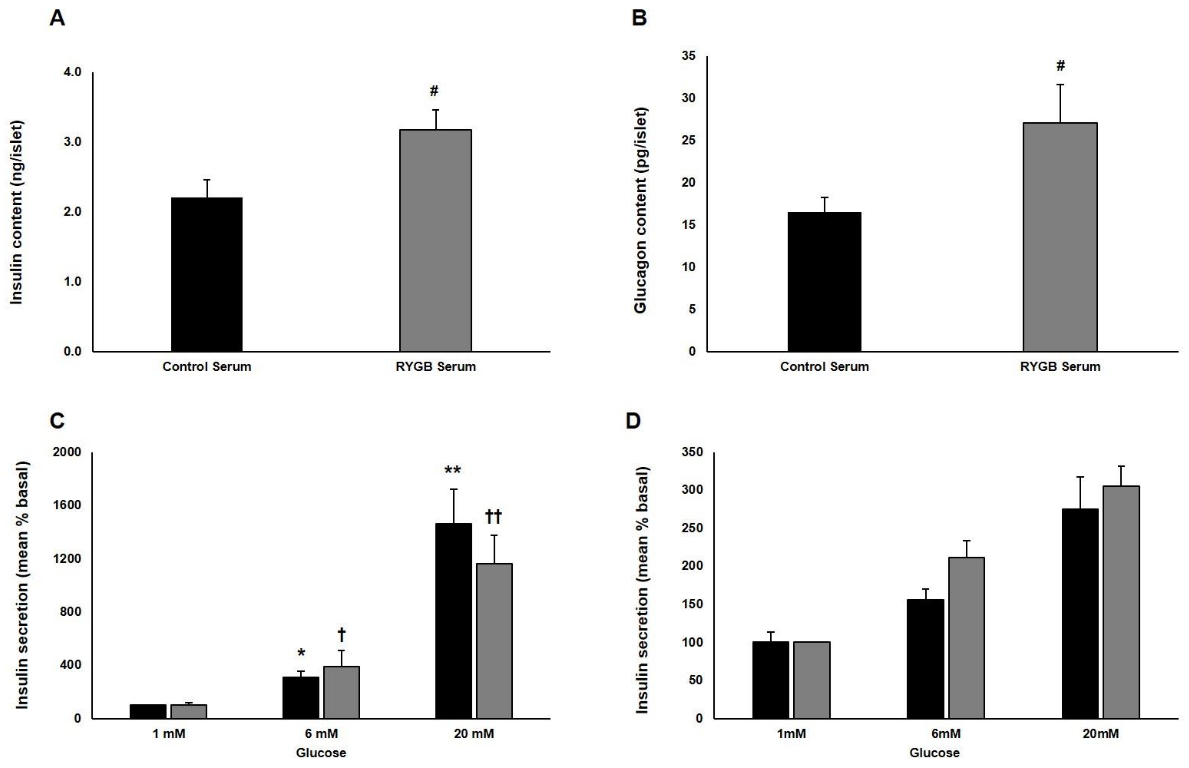

Figure S3. Related to Figure 2 and Figure 3. Islet function post-RYGB is modulated by a humoral factor, distinct from GLP-1.

(A-B) Insulin **(A)** and glucagon **(B)** content were measured in rat islets treated for 48 h with serum obtained from non-diabetic Wistar animals that had undergone sham (black) or RYGB surgery (grey bars). $^{\#}P<0.05$ vs control serum. Data represented as mean ± SEM (n=8 rats, 5-6 experiments per group).

(C) Islets from RYGB animals treated with glucose in the presence (grey) or absence (black bars) of the GLP-1 receptor antagonist exendin (9-39; 1µM). Secretion data are presented as per cent of that secreted at basal glucose (Sham: 0.023±0.004; RYGB: 0.046±0.001; n=3-4 experiments). $^{*}P<0.05$, $^{**}P<0.01$ vs 1mM glucose. $^{\dagger}P<0.05$, $^{\dagger\dagger}P<0.01$ vs 1mM glucose + exendin (9-39).

(D) Islets from non-diabetic Wistar rats were treated chronically with serum from GK bypassed animals in the presence (grey) or absence (black bars) of exendin (9-39; 1µM). Secretion data are presented as per cent of that secreted at basal glucose (1mM glucose alone: 0.375±0.049; +exendin (9-39): 0.399±0.001; n=3 experiments). Data represented as mean ± SEM.

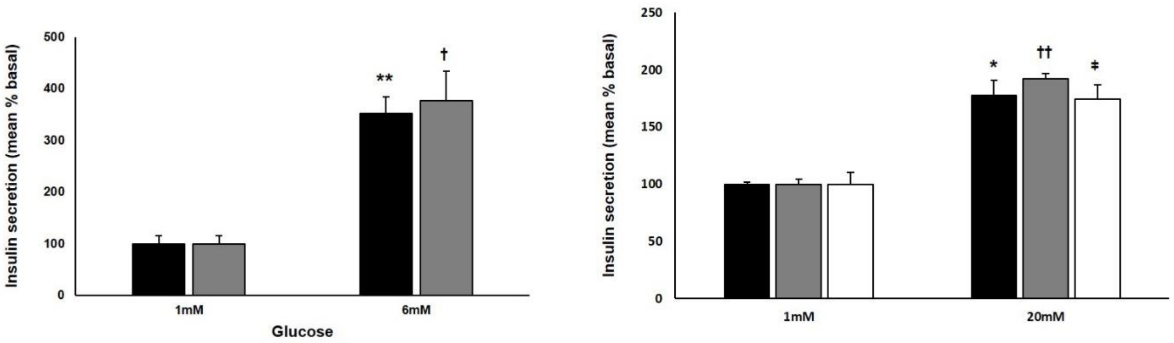

Figure S4. Related to Figure 3. Insulin secretion in not modulated by acute administration of PYY, or by exogenously applied compounds.

(A) Insulin secretion from rat islets treated acutely with (grey) or without (black) PYY. Secretion data are presented as per cent of that secreted at basal glucose (1mM glucose alone: 0.011±0.001; +PYY: 0.012±0.002; n=6 experiments). ***P*<0.01 vs 1mM glucose, †*P*<0.05 vs 1mM glucose with PYY. Data represented as mean ± SEM.

(B) Insulin secretion from rat islets treated chronically with PYY antibody (grey), BIBP-3226(white) or in the absence of either compound (black bars). Secretion data are presented as per cent of that secreted at basal glucose (1mM glucose alone: 0.105±0.002; +PYY antibody: 0.103±0.044; +BIBP-3226: 0.101±0.01; n=3-6 experiments).**P*<0.05 vs 1mM glucose, ††*P*<0.01 vs 1mM glucose with PYY antibody, ‡*P*<0.05 vs 1mM control with BIBP-3226.

Supplemental Reference

Chomczynski, P., and Sacchi, N. (1987). Single-step method of RNA isolation by acid guanidinium thiocyanate-phenol-chloroform extraction. Analytical biochemistry *162*, 156-159.

Pfaffl, M.W. (2001). A new mathematical model for relative quantification in real-time RT-PCR. Nucleic acids research *29*, e45.
